# Tumor M2-Pyruvate Kinase, Matrix Carbonic Anhydrase IX, and Metalloproteinase 9 — Novel Prognostic Markers of Renal Cell Carcinoma

**DOI:** 10.17691/stm2020.12.2.05

**Published:** 2020

**Authors:** Z.V. Amoev, A.V. Alyasova, T.N. Gorshkova, E.I. Samsonova, E.V. Strokina, K.N. Kontorshchikova

**Affiliations:** Urologist, Department of Urology No.2, Clinical Hospital No.1, Privolzhsky District Medical Center of Federal Medico-Biologic Agency of Russia, 14 Ilyinskaya St., Nizhny Novgorod, 603000, Russia;; Professor, Department of Oncology, Radiation Therapy and Radiology, Privolzhsky Research Medical University, 10/1 Minin and Pozharsky Square, Nizhny Novgorod, 603005, Russia;; Head of Clinical Diagnostic Laboratory, Privolzhsky District Medical Center of Federal Medico-Biologic Agency of Russia, 14 Ilyinskaya St., Nizhny Novgorod, 603000, Russia;; Physician, Clinical Diagnostic Laboratory, Privolzhsky District Medical Center of Federal Medico-Biologic Agency of Russia, 14 Ilyinskaya St., Nizhny Novgorod, 603000, Russia;; Physician, Clinical Diagnostic Laboratory, Privolzhsky District Medical Center of Federal Medico-Biologic Agency of Russia, 14 Ilyinskaya St., Nizhny Novgorod, 603000, Russia;; Professor, Head of the Department of Clinical Laboratory Diagnostics, Privolzhsky Research Medical University, 10/1 Minin and Pozharsky Square, Nizhny Novgorod, 603005, Russia

**Keywords:** tumor M2-pyruvate kinase, matrix carbonic anhydrase IX, metalloproteinase 9, renal cell carcinoma, markers of renal cell carcinoma

## Abstract

**The aim of the study** was to assess the possibility of using plasma levels of tumor M2-pyruvate kinase (Tu M2-PK), matrix carbonic anhydrase IX (CA9), and matrix metalloproteinase 9 (MMP9) in patients with renal cell cancer as predictors of the disease course and the response to treatment.

**Materials and Methods:**

Samples of blood plasma or serum of 46 patients with clear cell renal cancer T_1–4_N_0–1_M_0–1_ obtained before surgery and 8–9 days after surgery were tested. The control group consisted of 20 practically healthy individuals, comparable in age with the examined patients. Quantitative determination of Tu M2-PK in EDTA-added blood plasma was performed by enzyme-linked immunosorbent assay using a ScheBo Tumor M2-PK test (Germany). Determination of CA9 by ELISA was performed using a Human Carbonic Anhydrase IX Quantikine ELISA Kit (USA) and MMP9 — using a Quantikine ELISA Kit (USA).

**Results:**

In patients with renal cell carcinoma, a statistically significant increase in the level of Tu M2-PK, CA9 and a statistically significant decrease in MMP9 in comparison with the control group were found. The level of Tu M2-PK in patients with localized kidney cancer was significantly lower than in patients with disseminated cancer. An increase in size of the primary tumor and a decrease in the degree of its differentiation correlated with an increase in Tu M2-PK, and decrease in CA9 and MMP9 in the blood serum. Performing surgery equivalent to nephrectomy did not change the Tu M2-PK levels in the early postoperative period, but caused a decrease in the levels of CA9 and MMP9.

**Conclusion:**

The results indicate a potential significance of Tu M2-PK, CA9, and MMP9 as biological markers for predicting the disease course in patients with renal cell carcinoma.

## Introduction

The difficulties of early diagnosis of renal cell carcinoma (RCC) and prediction of RCC dissemination necessitate a search for new prognostic markers [1]. One of possible candidates is tumor M2-pyruvate kinase (Tu M2-PK), one of the known metabolic tumor markers. Elevated levels of this protein were detected in patients with malignant tumors of the gastrointestinal tract, kidneys, breast, and lung [2–4].

Matrix carbonic anhydrase IX (CA9) is a transmembrane glycoprotein from the carbonic anhydrase group of enzymes that play an important role in the regulation of proton secretion and pH stability in the cell [5, 6]. CA9 expression is detected in almost all types of tumors (cancer of cervix, esophagus, lung, breast, brain, or vulva). In recent years, a number of studies reported on the predictive value of CA9 expression in clear RCC [7, 8]. However, no detailed study on the levels of CA9 in the blood serum of patients with RCC and its dynamics was presented.

Matrix metalloproteinases (MMPs) are a family of endopeptidases expressed at various stages of tumor development and involved in the pathways that allow malignant cells to avoid immune response and apoptosis [9, 10]. A high level of MMP9 expression was detected in tumor cells in cancer of the lung, bladder, cervix, rectum, prostate, kidney, and the endometrium [10–12]. Yet, we found no reports on correlations between the above marker proteins and clinical and morphological characteristics of RCC, or its response to treatment.

**The aim of the study** was to assess the possibility of using plasma levels of tumor M2-pyruvate kinase (Tu M2-PK), matrix carbonic anhydrase IX (CA9), and matrix metalloproteinase 9 (MMP9) in patients with renal cell cancer as predictors of the disease course and the response to treatment.

## Materials and Methods

The study was performed using samples of blood plasma or serum from 46 patients (average age 58.3 years) with clear cell renal cancer T_1–4_N_0–1_M_0–1_, obtained before and 8–9 days after surgery. In 33% of patients (15/46), the disease was staged as T_1_, in 26% (12/46) — T_2_, in 28% (13/46) — T_3_, and in 13% (6/46) — T_4_. Localized forms of the disease (T_1–3_N_0_M_0_) was detected in 46% of patients (21/46), and disseminated forms — in 54% (25/46).

The diagnosis and treatment procedures were in line with the recommendations for the diagnosis and treatment of malignant neoplasms, approved by the Ministry of Health of the Russia. In all patients, the diagnosis of clear cell renal cancer was confirmed by histological examination. The degrees of tumor differentiation according to Fuhrman nuclear grade were as follows: grade I — 20% (9/46) patients, grade II — 26% (12/46), and grade III — 54% (25/46). All patients underwent nephrectomy. As a control, we used blood plasma or serum samples of 20 practically healthy individuals, comparable in age with the examined patients.

The study was conducted in accordance with the Helsinki Declaration (2013) and approved by the Ethics Committee of the Privolzhsky Research Medical University. Informed consent was obtained from each patient.

Quantitative determination of the metabolic tumor marker — Tu M2-PK in EDTA blood plasma by ELISA was performed using a ScheBo Tumor M2-PK test (ScheBo Biotech AG, Germany). To determine CA9 in the serum, we used a Human Carbonic Anhydrase IX Quantikine ELISA Kit (R&D Systems, USA); for MMP9 — a Quantikine ELISA Kit (R&D Systems, USA) was used.

**Statistical processing** was performed using the Statistica v. 6.0 software package. To test the hypothesis that the data set fitted the normal distribution, the Shapiro–Wilk test was used. The results are presented in the form M±σ, where M is the mean value, and σ is the standard deviation. To assess significance of the differences between two samples, t-test was used. Differences were considered statistically significant at p<0.05. The significance of the differences between fractions was calculated by the Fisher transformation method.

## Results and Discussion

**Figure 1:**
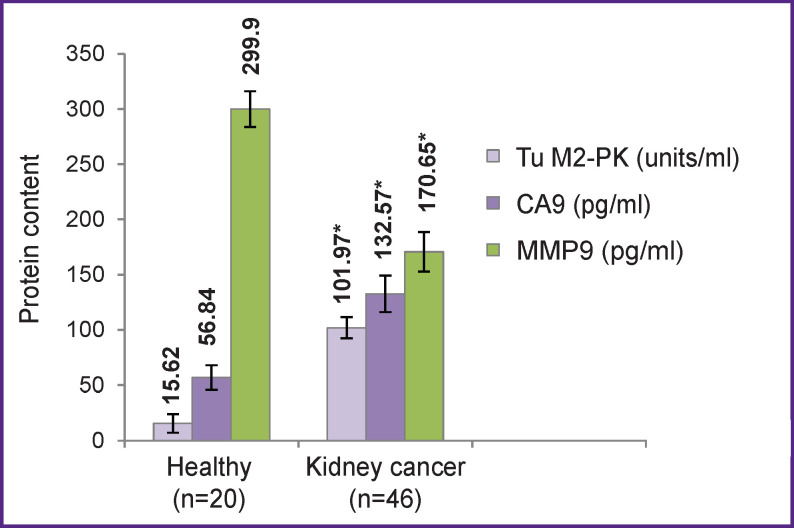
Levels of Tu M2-PK, CA9, and MMP9 in blood samples of healthy individuals and patients with kidney cancer * Differences from control are statistically significant (p<0.05)

According to the obtained results, patients with RCC have significantly higher levels of Tu M2-PK, and CA9 and a significantly lower level of MMP9 than the RCC-free controls (Figure 1).

The increase in Tu M2-PK in the blood plasma may be due to specifics of metabolism in tumor cells and their accelerated proliferation compared to normal cells. The increase in the serum concentration of CA9 may be due to tissue hypoxia — the mechanism underlying the overexpression of CA9 in tumors tissues. In hypoxia, the hypoxia-induced factor (HIF-1) stimulates additional transcription of the CA9 gene. CA9 promotes a local increase in acidity by tumor cells, which leads to tumor growth and metastases. Another mechanism that regulates the increased expression of CA9 in tumor tissues is the loss of the Hippel–Lindau tumor suppressor gene (*VHL*). Mutations of the *VHL* gene can lead to overexpression of CA9 in tumor cell lines. According to [7], high expression of CA9 is associated with a favorable prognosis for localized and metastatic RCC and good relapse-free survival. According to Bui et al. [13], a high expression of CA9 suggests better survival even after provision for T-stage, Fuhrman nuclear grade, and clinical condition are made (p≤0.005).

Metalloproteinases are considered in the literature as molecules crucial for the process of tumor metastasis [9]. Being the key enzymes of the connective tissue, they participate in pathological processes that involve proliferation, cell migration, and extracellular matrix restructuring. The significant decrease in MMP9 found in the serum of RCC patients can be associated with increased utilization of MMP9 in the tissue remodeling, mobilization of matrix-related growth factors and processing of cytokines typical of tumor growth. In addition, some authors consider MMP9 as a critical causative factor (trigger) of tumor-induced angiogenesis [14].

**Figure 2:**
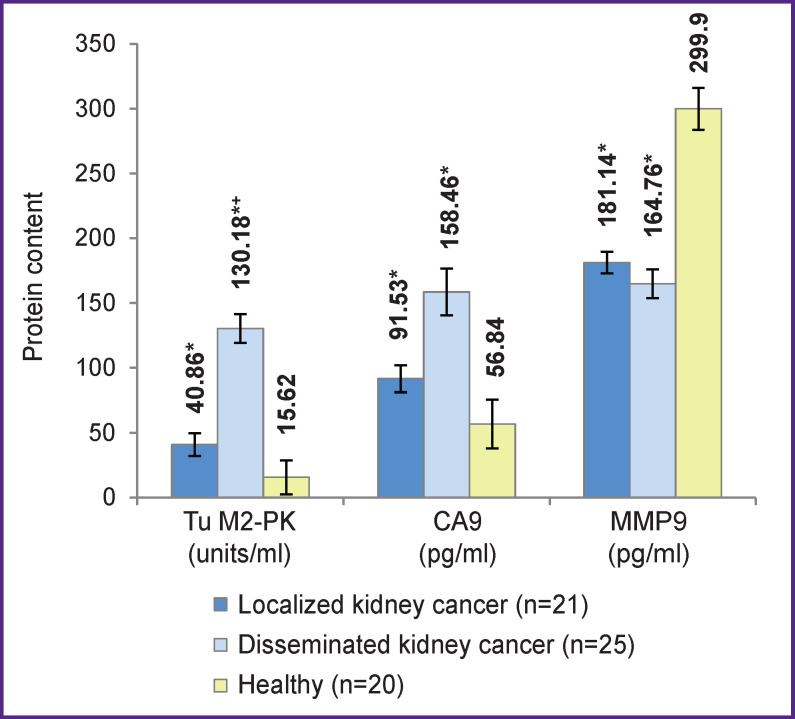
Levels of Tu M2-PK, CA9, and MMP9 in patients with localized and disseminated kidney cancers * Differences from control are statistically significant (p<0.05); ^+^ differences from the group with localized tumors (p<0.05)

**Figure 3:**
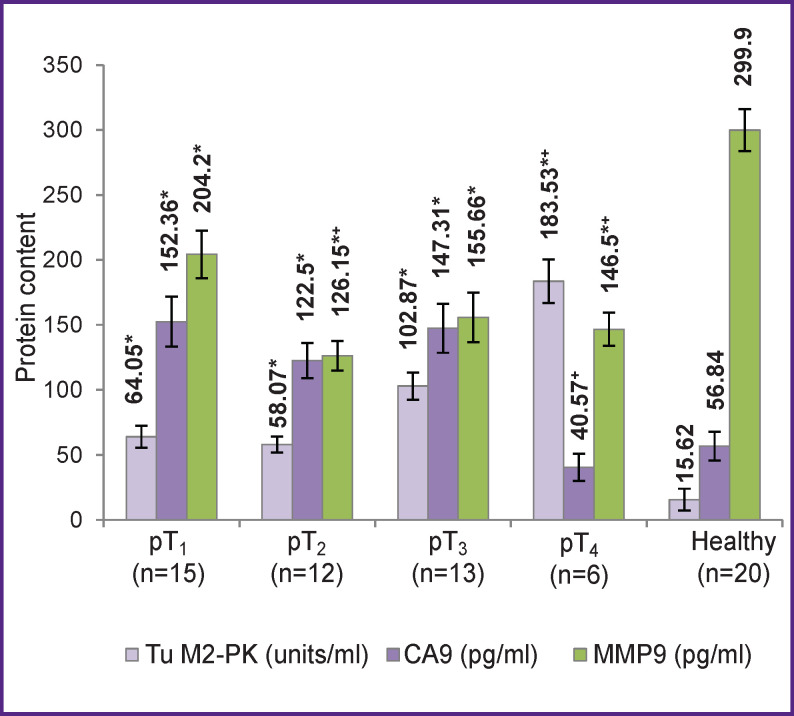
Levels of Tu M2-PK, CA9, and MMP9 in blood samples of patients with different sizes of primary tumor * Differences from control are statistically significant (p<0.05); ^+^ differences from the group with pT1 tumors (p<0.05)

We then compared the levels of Tu M2-PK, CA9, and MMP9 in blood samples of patients with either localized or disseminated forms of the disease (Figure 2), as well as correlations between these levels and the size of the primary tumor (Figure 3).

This, in patients with localized kidney cancer, the plasma levels of Tu M2-PK were significantly higher than normal, which could be due to impaired cell metabolism affected by tumor growth; in addition, these levels were significantly lower (3.2-fold) than those in patients with disseminated forms of RCC. Concentrations of CA9 did not significantly differ between these two forms of cancer and remained significantly higher than the respective values in healthy individuals. The serum levels of MMP9 did not significantly differ between the groups with localized and disseminated RCC, remaining significantly lower than that in control.

With an increase in size of the primary tumor to pT_4_, we observed a statistically significant increase in the plasma level of Tu M2-PK as compared to patients with pT_1_ tumors (p<0.05). Other researchers [4] also noted a similar increase as early as at stage I and a further increase along with disease progression. The concentration of CA9 in the blood serum of patients with pT_1_–T_3_ tumors was significantly higher than normal; however, in patients with pT_4_ tumors it was again close to normal and thus lower than in cases of pT_1_ tumors (p<0.05). It is known that the loss of CA9 expression is an unfavorable prognostic factor associated with the development of metastases and relapse of the disease, as well as a low rate of disease-free survival [7, 15]; in most cases of CA9 loss, the disease is metastatic already at the time of diagnosis. Indeed, in our study in patients with pT_4_ tumors, distant metastases were detected during the primary patient examination. An increase in size of the primary tumor was accompanied by a decrease in the amount of MMP9 in the blood serum. In individuals with tumors classified as pT_4_, the level of MMP9 was 1.4 times lower than that in patients with pT_1_ tumors (p<0.05).

**Figure 4:**
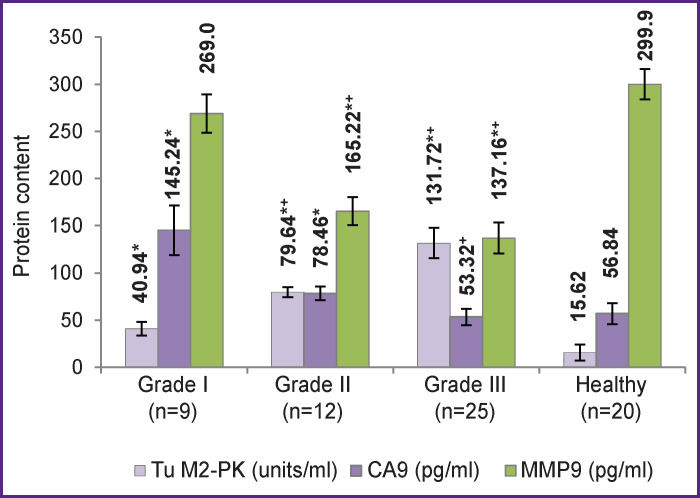
Levels of Tu M2-PK, CA9, and MMP9 in blood samples of patients with various degrees of tumor differentiation * Differences from control are statistically significant (p<0.05); ^+^ differences from the group with tumors of grade I malignancy (p<0.05)

A statistically significant increase in the level of Tu M2-PK was noted with a decrease in the degree of RCC differentiation (Figure 4); that might reflect increasing metabolic disturbances in patients with a more aggressive course of RCC. Thus, in patients with grade III tumor differentiation, the level of Tu M2-PK in the blood plasma was 3.2-fold higher (p<0.05) than that in patients with grade I tumor differentiation.

In contrast, the presence of CA9 in the serum significantly decreased with de-differentiation of tumor cells. In patients with tumors differentiated at grade III, the CA9 levels did not differ from the norm and were 2.7 times lower (p<0.05) than those in patients with tumors differentiated at grade I. It is known that with a decrease in tumor differentiation, the expression of CA9 is often reduced and even completely lost. Shchukin et al. [16] showed a correlation between CA9 expression and tumor differentiation (Fuhrman nuclear grade); however, other researchers did not confirm this relationship [8]. According to some authors [10, 15], low expression of CA9 is associated with low cancer-specific survival, overall survival, and progression-free survival.

The concentration of MMP9 in the blood serum of patients with poorly differentiated tumors also decreased (statistically significant) compared not only to control, but also to those with well-differentiated tumors. Probably, such changes are associated with increased consumption of the MMP9 protein in actively proliferating cells; in these cells, MMP9 destroys collagen IV of the basement membrane and activates the invasion of tumor cells into the underlying tissues. Avdoshin et al. [17] reported differences between urothelial carcinomas of low and high malignancy in terms of expression of MMP9. It should be noted that the decrease in serum MMP9 with increasing tumor sizes or with decreasing tumor differentiation does not corroborate with the expression of MMP9 in tumor cells as reported by others [10]. Despite the increased proliferation of tumor cells together with MMP9 production, a significant part of MMP9 may still be utilized locally within the malignant cells (for proteolysis of membrane proteins, invasion of tumor cells into the underlying tissues, or neo-angiogenesis) and not reach the bloodstream.

In the postoperative period, the level of Tu M2-PK in the blood plasma did not differ from the pre-operative level; the similar result was previously reported by other researchers [4]. An individual analysis showed that the content of Tu M2-PK decreased twofold from the baseline in only 9% of patients. In other cases, its concentration either did not change or increased 1.5–1.8-fold from the preoperative value. According to report [18], the post-surgery level of Tu M2-PK returned to normal after around 11 weeks, that was later than in our study; the normalization may be preceded by an initial increase in the level of Tu M2-PK in the blood.

The concentration of CA9 after surgery decreased by 2.17 times as compared with the initial level (before surgery — 134.53±15.29, after surgery — 61.78±22.06 pg/ml; p<0.05) and did not differ from the norm (56.84±11.10 pg/ml). An individual analysis revealed a decrease in CA9 by 2.5–5.5 times in 70% of the examined patients on the 8–9^th^ day after the radical operation. These results confirm the existing assumption [10, 13] about the predominant production of CA9 by tumor cells.

The content of MMP9 after surgery did not change, remaining significantly lower than that in the control group. An individual analysis revealed that in all examined patients, the level of MMP9 in the blood serum on the 8–9^th^ day after surgery had a decreasing trend; however, a pronounced decrease in the level of MMP9 by 1.5–2 times was observed only in 40% cases.

Thus, the level of Tu M2-PK in the blood plasma of patients with RCC reflects the spread and activity of the tumor process. This marker does not change in early stages after surgery.

The level of CA9 in the blood serum of patients with kidney cancer is significantly higher than that in healthy individuals; yet it is significantly lower in patients with pT_4_ tumors compared to patients with tumors of smaller sizes, as well as in patients with low-differentiation tumors compared to those with highly differentiated neoplasms. Serum CA9 levels are significantly reduced in the postoperative period.

The concentration of MMP9 in the blood serum of RCC patients significantly decreases with increasing aggressiveness of the tumor process and remains so in the early postoperative period.

## Conclusion

The presented results strongly indicate the potential significance of the Tu M2-PK, CA9, and MMP9 proteins as markers capable of predicting the disease course in patients with renal cell carcinoma.
